# Supplementary material for: Concurrent optoacoustic tomography and magnetic resonance imaging of resting-state functional connectivity in the mouse brain
Source: Nat Commun. 2024 Dec 30;15:10791. doi: 10.1038/s41467-024-54947-y (PMC11685406; doi:10.1038/s41467-024-54947-y)
Supplement: Supplementary file 1 — Supplementary Figs. 1-23 and Table 1 [file 41467_2024_54947_MOESM1_ESM.pdf]

Supplementary Information

Concurrent optoacoustic tomography and magnetic resonance imaging of resting-state functional connectivity in the mouse brain

Irmak Gezginer[1,2,†], Zhenyue Chen[1,2,3,†], Hikari A. I. Yoshihara[1,2], Xosé Luís Deán-Ben[1,2], Valerio Zerbi[4,5], and Daniel Razansky[1,2,*]

[1]Institute for Biomedical Engineering and Institute of Pharmacology and Toxicology, Faculty of Medicine, University of Zurich, Switzerland

[2]Institute for Biomedical Engineering, Department of Information Technology and Electrical Engineering, ETH Zurich, Switzerland

[3]Institute of Precision Optical Engineering, School of Physics Science and Engineering, Tongji University

[4]Department of Psychiatry, Faculty of Medicine, University of Geneva, Switzerland

[5]Department of Basic Neurosciences, Faculty of Medicine, University of Geneva, Switzerland

*Correspondence

Daniel Razansky, Institute for Biomedical Engineering, Wolfgang-Pauli-Str. 27, 8093 Zurich, Switzerland
Email: daniel.razansky@uzh.ch

[†]These authors contributed equally: Irmak Gezginer, Zhenyue Chen

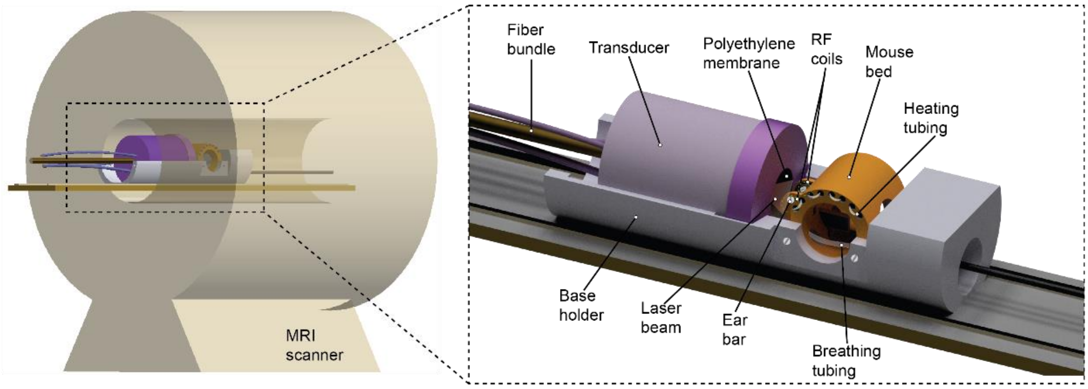

Supplementary Figure 1. Layout of the MROT scanner enabling simultaneous MRI and OAT readings from the murine brain. The main components of the OAT module are shown in the inset.

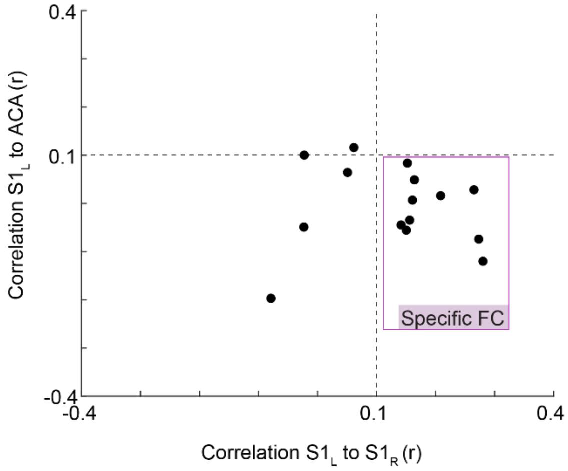

Supplementary Figure 2. Functional connectivity specificity was assessed for each fMRI dataset by examining the concurrent presence of strong (r> 0.1) inter-hemispheric connectivity in sensory areas, along with either weak connectivity or anti-correlation (r< 0.1) between the sensory areas and the anterior cingulate area. 10 datasets meeting these criteria were included in further analysis.

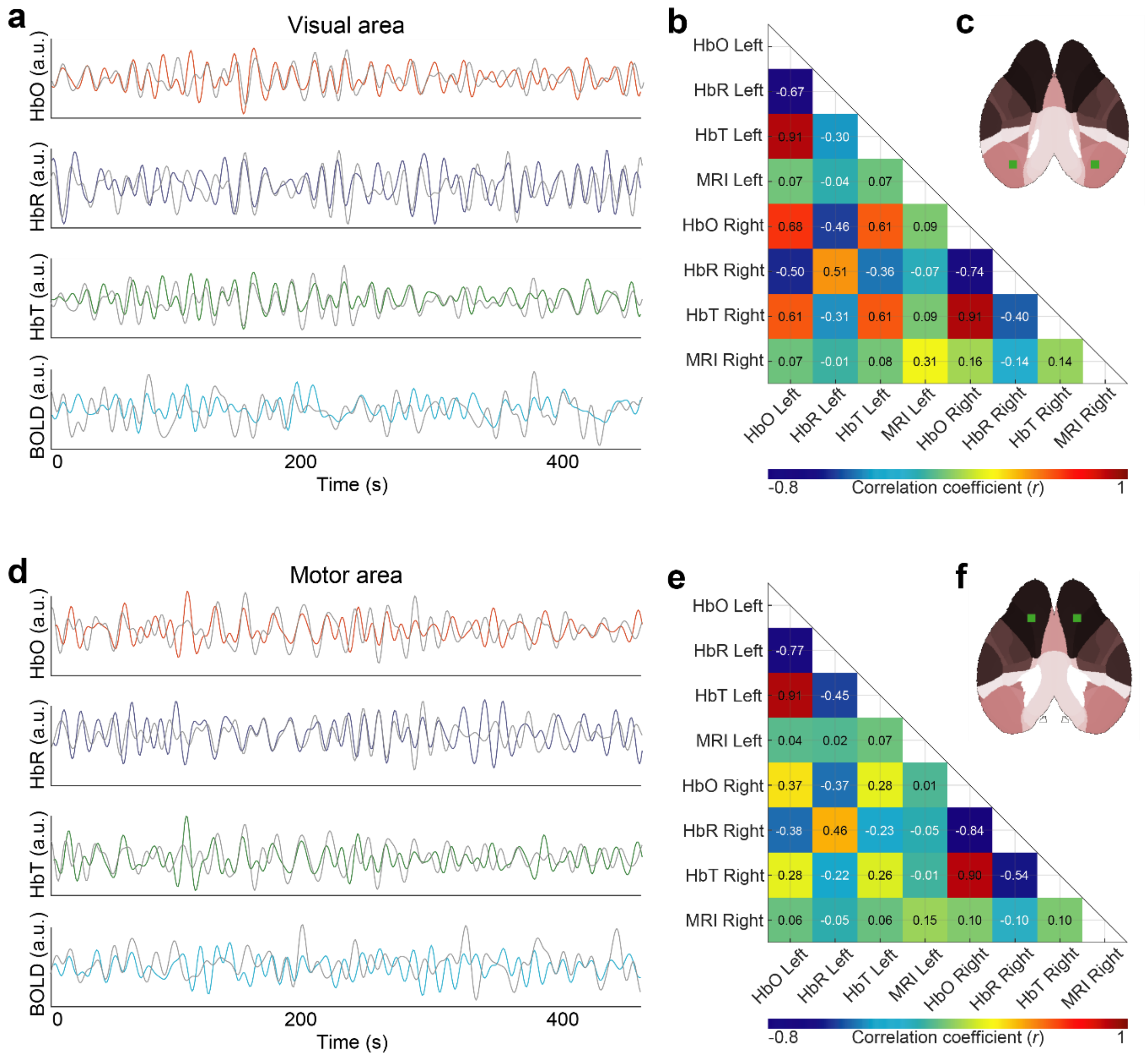

Supplementary Figure 3. Representative HbO, HbR, HbT, and BOLD pre-processed time-courses (a, d) and their corresponding correlations (b, e) from the visual (c) and motor (f) areas of a selected subject. **(a, d)** The average time-courses for each hemodynamic channel were plotted from ROIs located in the left hemisphere, with the corresponding right hemisphere time-courses shown in gray. **(b, e)** Correlation matrices were generated by calculating Pearson's correlation coefficients across the signals. **(c, f)** The ROI locations are highlighted in green, overlaid on the Allen mouse brain atlas.

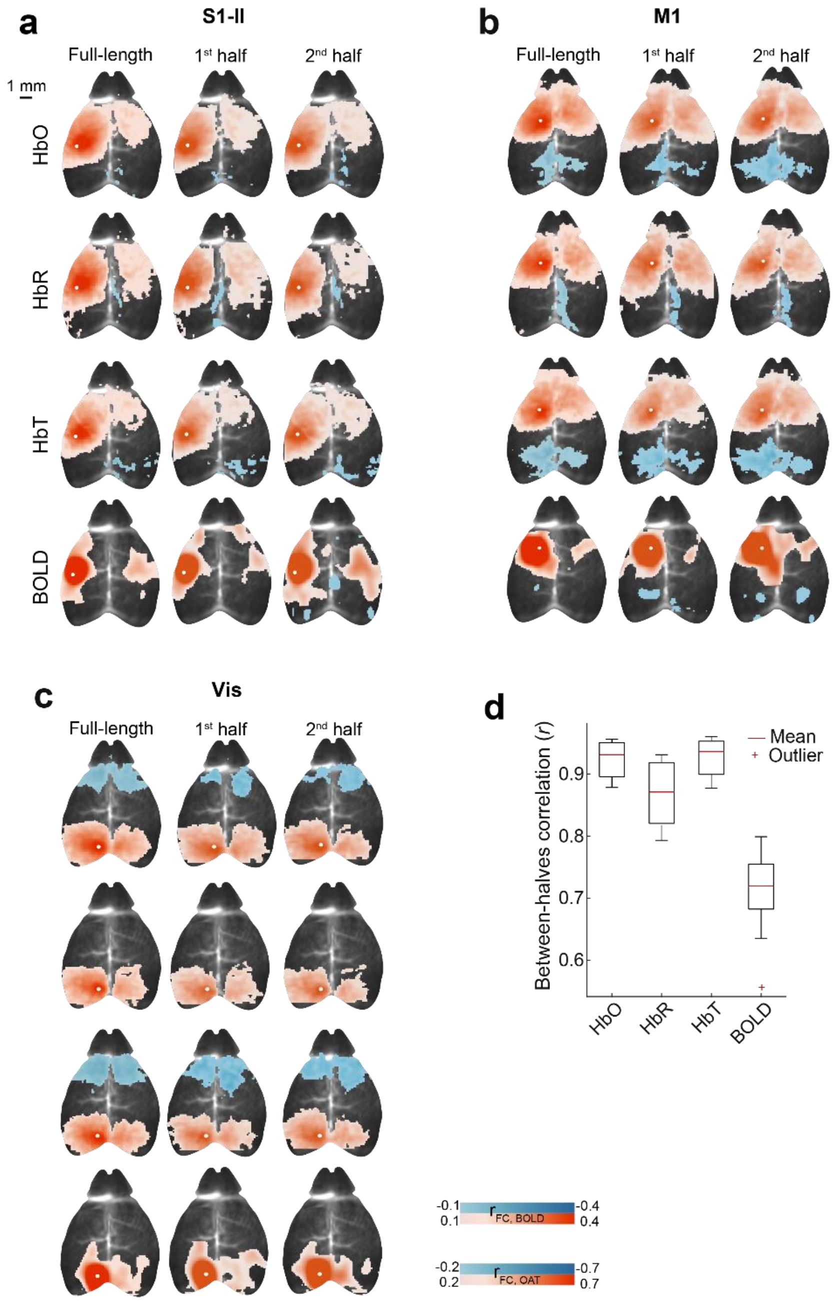

Supplementary Figure 4. (a-c) Group seed-maps derived from HbO, HbR, HbT, and BOLD for the somatosensory (a), motor (b), and visual (c) regions in the left hemisphere. Seed-maps were generated for each region using the full dataset, as well as the first and second halves of the data. **(d)** The correlation between rsFC maps derived from the first and second halves was computed across the functional regions defined in Fig. 2, incorporating a total of 14 seeds. S1-ll, primary somatosensory area, lower limb; M1, primary motor cortex; Vis, primary visual area. The central line within the box represents the mean, while the lower and upper box edges denote the 25th and 75th percentiles, respectively. The whiskers extend to the furthest data points within the non-outlier range.

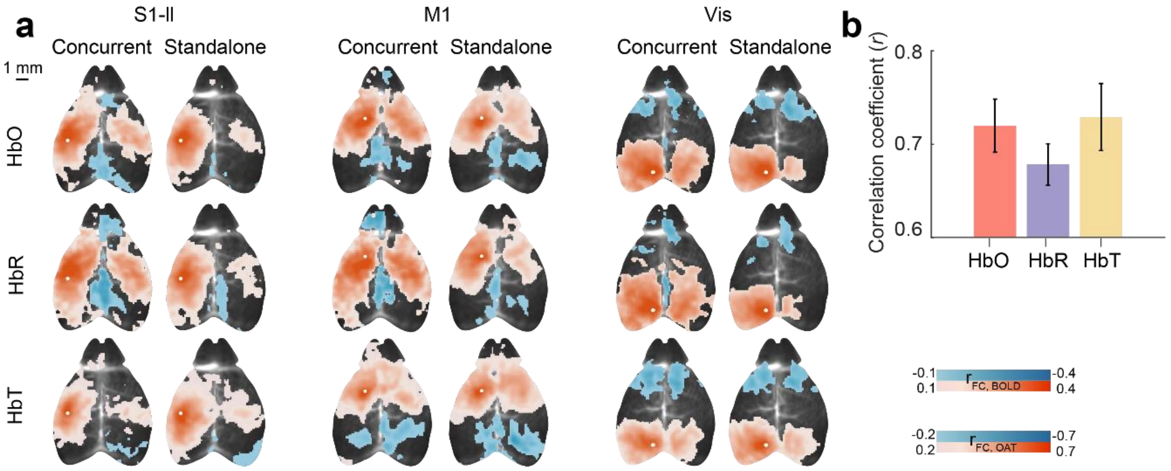

Supplementary Figure 5. (a) fOA seed-maps from a representative mouse, comparing those obtained during concurrent MROT imaging with those from fOA imaging conducted independently, without the influence of magnetic gradients. **(b)** Correlation analysis of the seed-maps (14 maps per component) between the concurrent and standalone acquisitions for each hemodynamic component. S1-ll, primary somatosensory area, lower limb; M1, primary motor cortex; Vis, primary visual area. Data are presented as mean values +/- SEM.

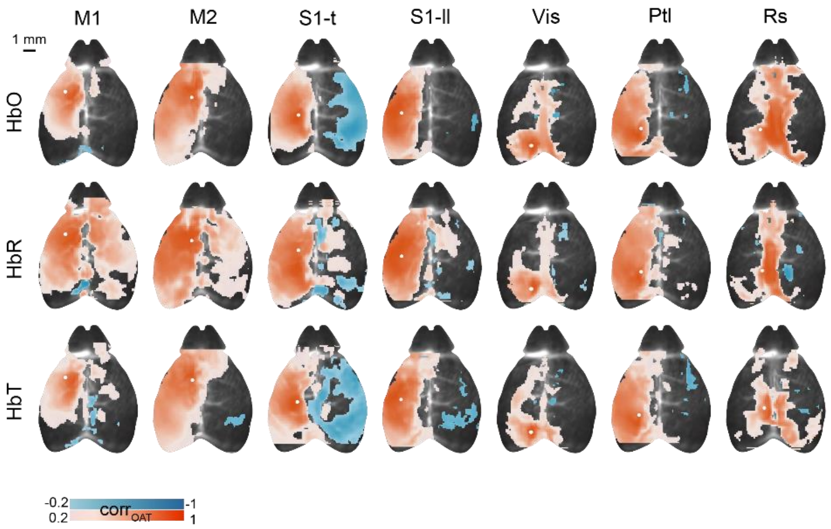

Supplementary Figure 6. Seed-maps of HbO, HbR, and HbT derived from a representative mouse utilizing filtered backprojection reconstruction. The entire dataset underwent preprocessing, denoising, and seed-based analysis identical to the procedures employed with the data presented in Fig. 2a, utilizing model-based reconstruction. Filtered backprojection resulted in non-local rsFC across all components, showing elevated non-specific intrahemispheric connectivity. This is likely due to higher noise levels and the presence of negativity artifacts, which are common in backprojection-based methods. M1, primary motor cortex; M2, secondary motor cortex; S1-t, primary somatosensory area, trunk; S1-ll, primary somatosensory area, lower limb; Vis, primary visual area; Ptl, parietal area; Rs, Retrosplenial area.

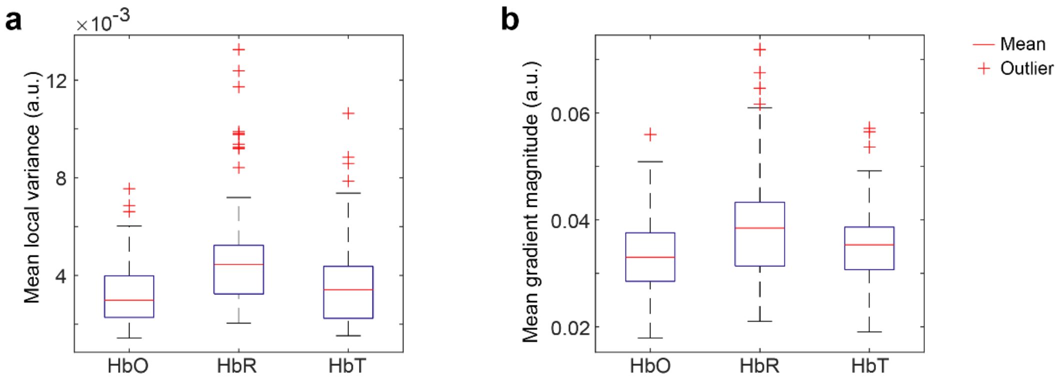

Supplementary Figure 7. Mean local variance (a) and mean gradient magnitude (b) for HbO, HbR, and HbT seed functional connectivity maps. These metrics were calculated across 14 seed locations per subject, yielding 140 data points per distribution (n= 140). The central line within the box represents the mean, while the lower and upper box edges denote the 25th and 75th percentiles, respectively. The whiskers extend to the furthest data points within the non-outlier range.

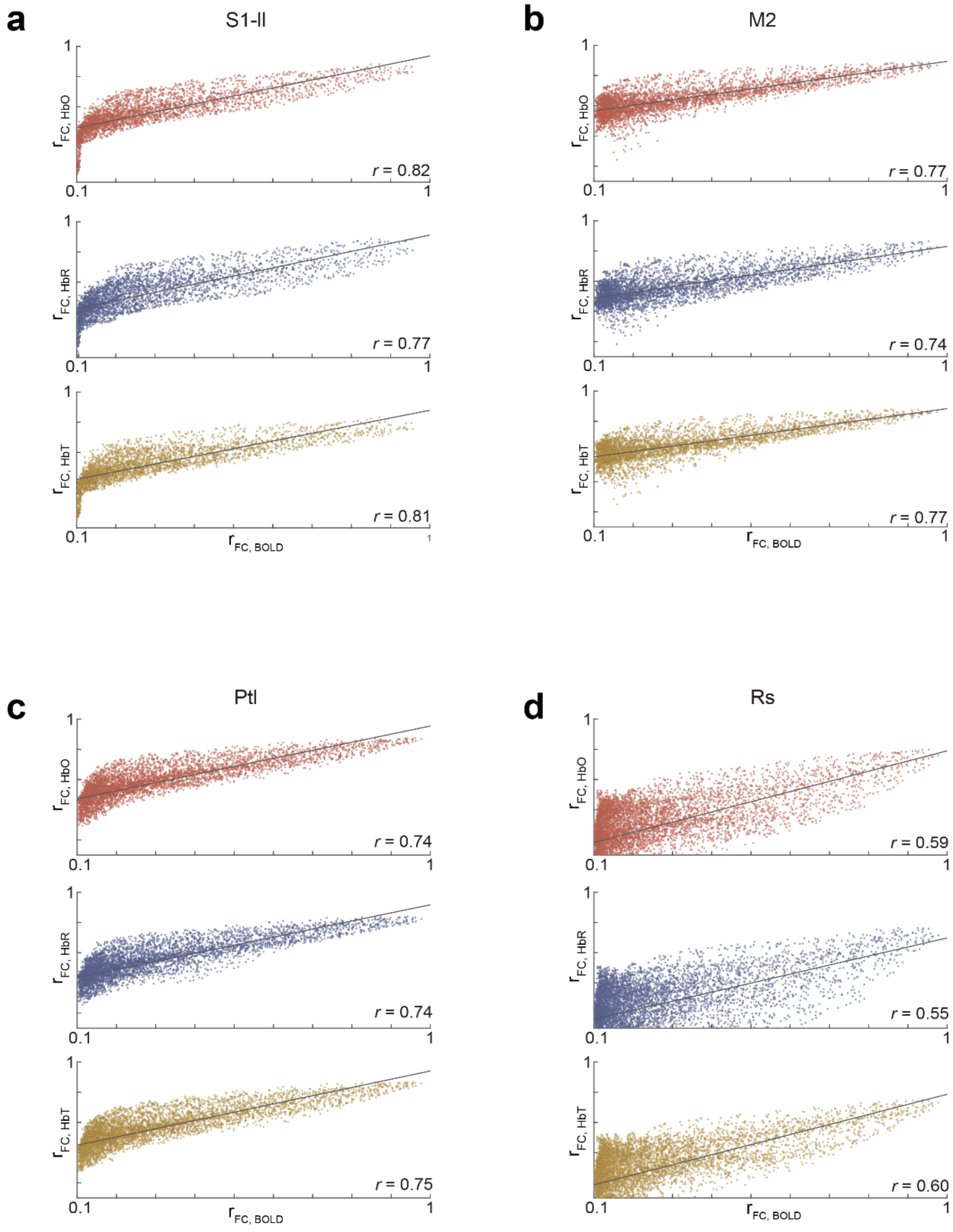

Supplementary Figure 8. Voxel-wise relationship of connectivity derived from group seed-maps of the remaining seeds (i.e. primary somatosensory area- lower limb (a), secondary motor area (b), parietal area (c), and retrosplenial area (d) seeds) located on the left hemisphere.

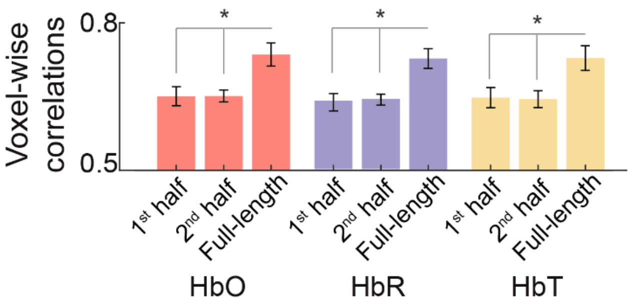

Supplementary Figure 9. Average voxel-wise correlation coefficients computed from HbO, HbR, and HbT using the first half, second half, and full length of the datasets. The analysis was conducted similarly to Fig. 2c-e. Voxel-wise correlations of FC (r > 0.1) were calculated from group seed-maps of the fourteen defined seeds. (*) $p< 0.05$, paired t-test. Data are presented as mean values +/- SEM.

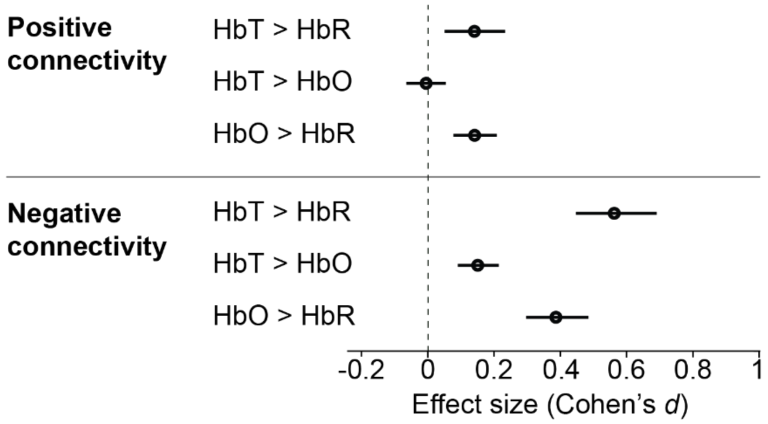

Supplementary Figure 10. The effect sizes, accompanied by 95% confidence intervals, depict the differences in correlation strengths compared to BOLD functional connectivity. In both positive and negative connections, HbT and HbO exhibited notably stronger correlations with BOLD functional connectivity in comparison to HbR. Notably, HbT displayed the highest correlation among the OAT components for both positive and negative connections, showcasing its superior association with BOLD functional connectivity. Data are presented as mean values and 95% confidence intervals.

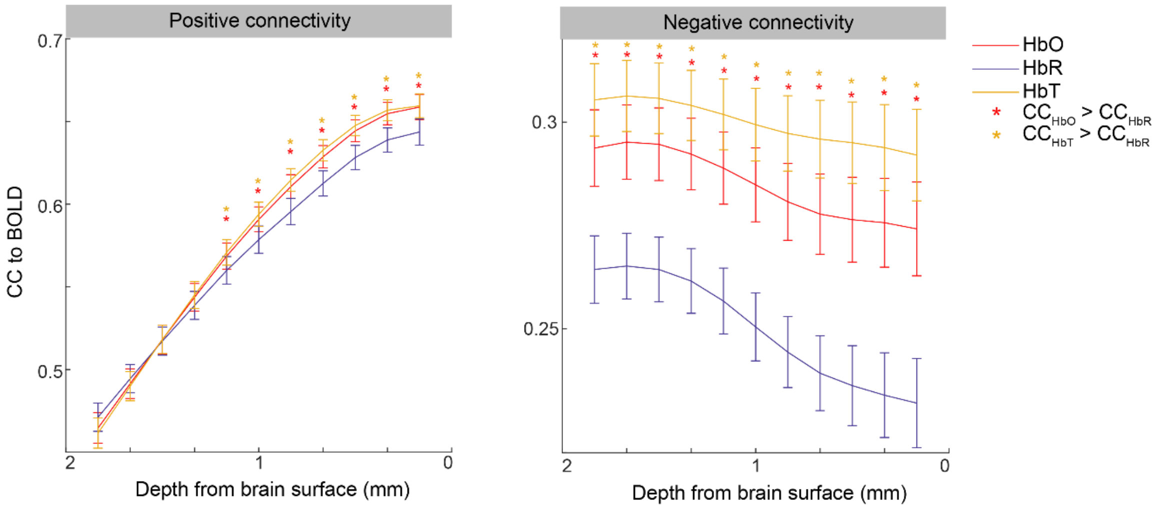

Supplementary Figure 11. Cross-correlation of fOA and BOLD seed-maps across different depths. Volumes with a thickness of 3 voxels were centered at varying depths to calculate the cross-correlation of positive and negative connections. Stars denote significantly higher correlations (paired t-test) for HbO (red) or HbT (yellow) compared to HbR. Data are presented as mean values +/- SEM.

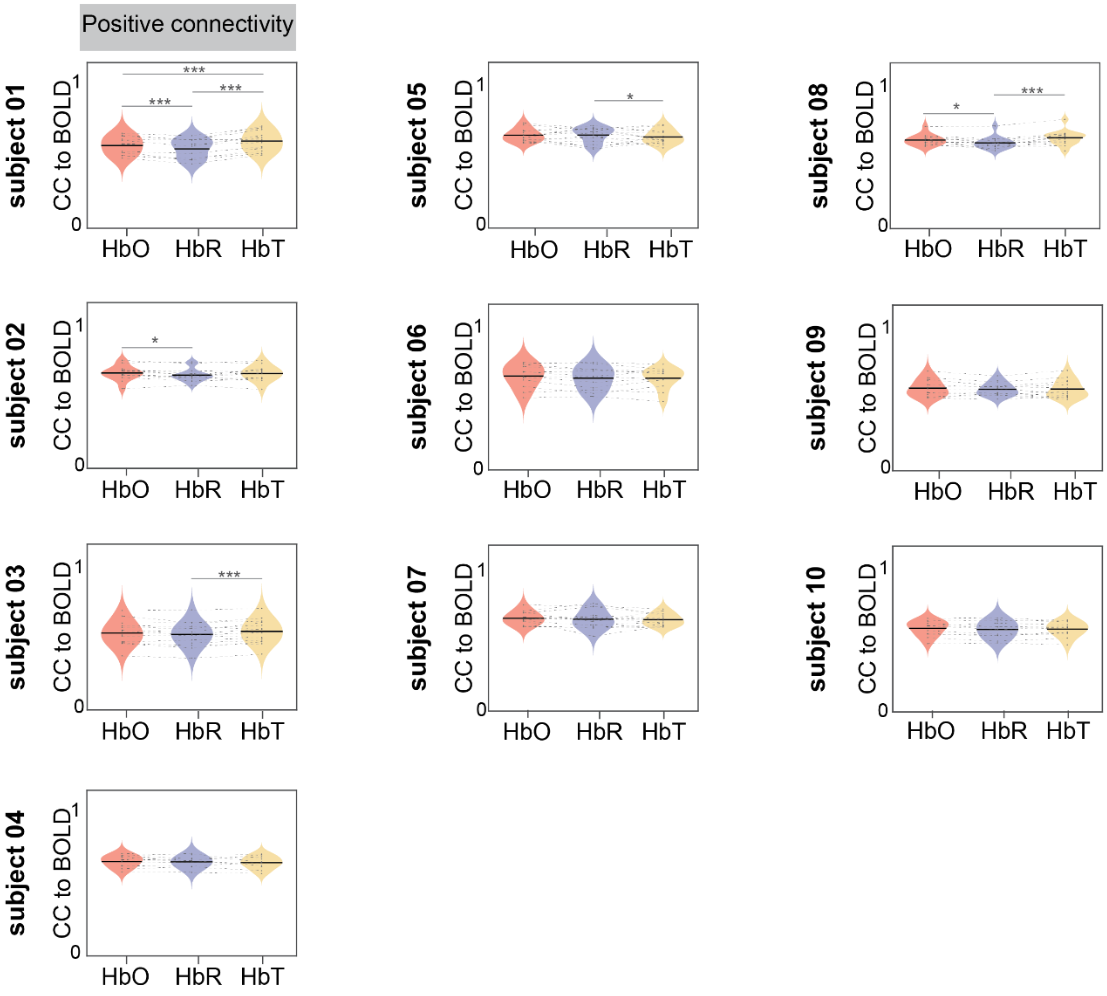

Supplementary Figure 12. Cross-correlation analysis of positive correlations within BOLD and OAT seed-based maps across all subjects. (*) $p< 0.05$, (**) $p< 0.01$, (***) $p< 0.001$, paired t-test.

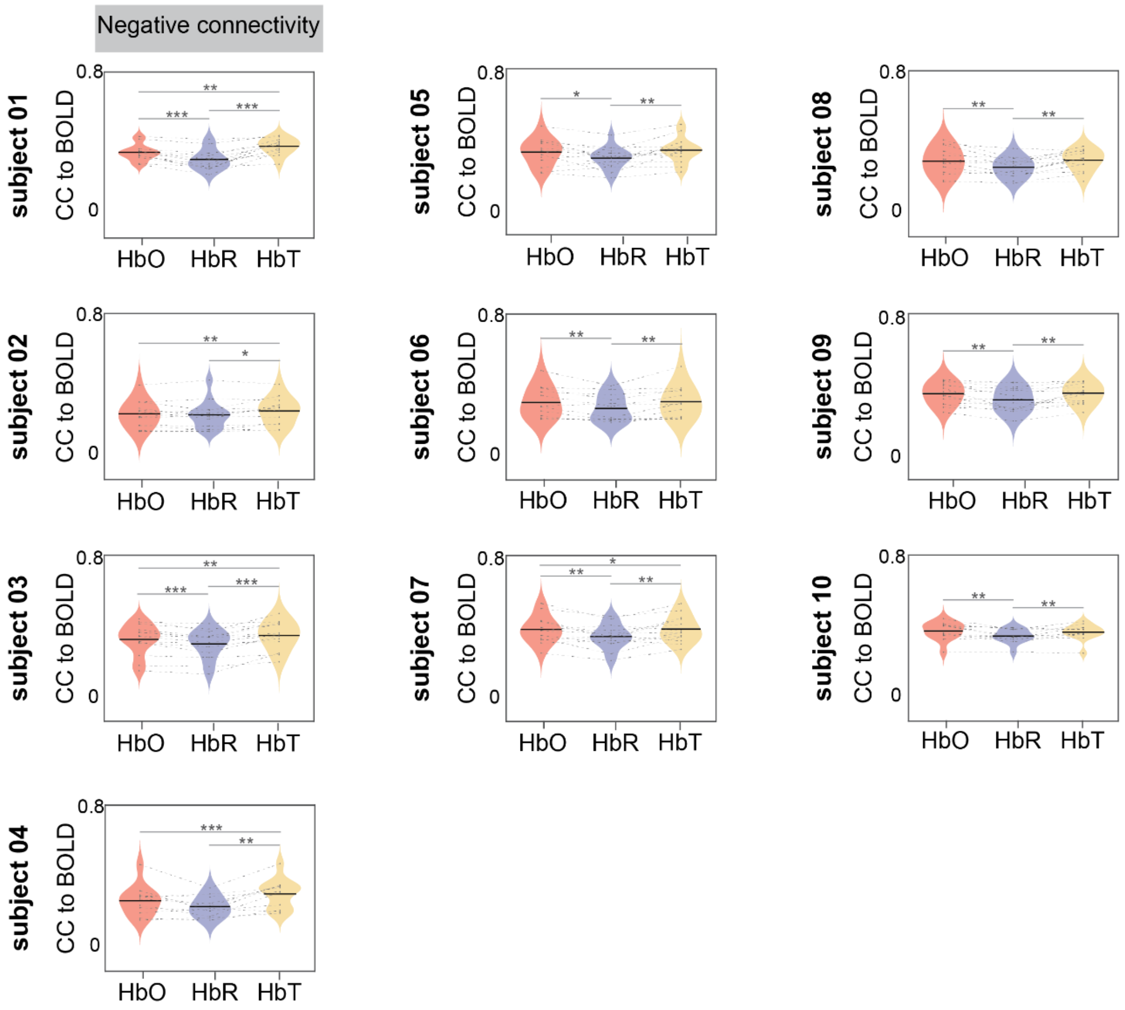

Supplementary Figure 13. Cross-correlation analysis of negative correlations within BOLD and OAT seed-based maps across all subjects. (*) $p< 0.05$, (**) $p< 0.01$, (***) $p< 0.001$, paired t-test.

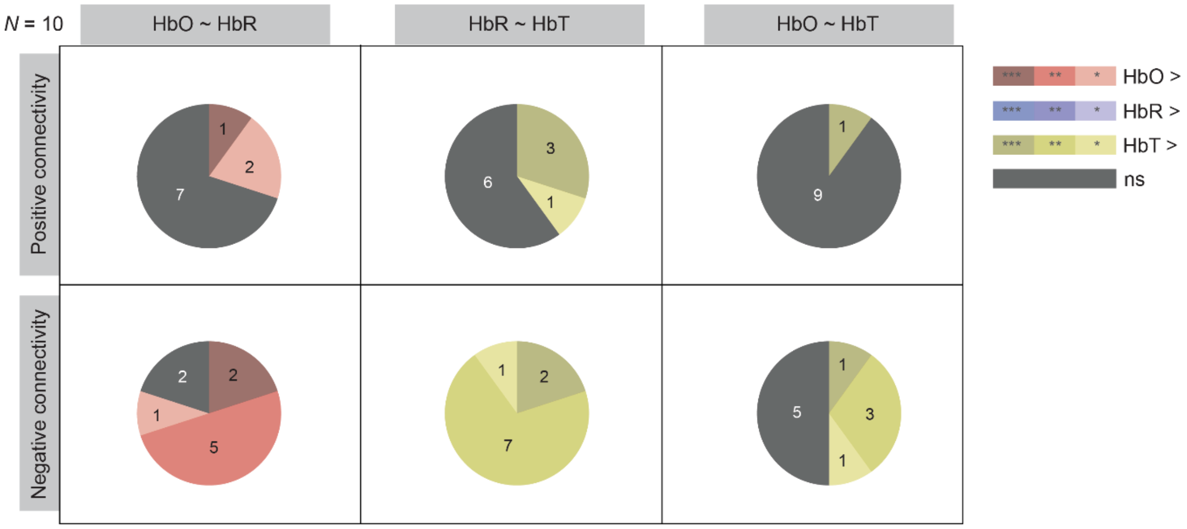

Supplementary Figure 14. Pie charts displaying subject counts with significantly higher correlations to BOLD across pairwise hemoglobin-components, differentiating positive and negative connectivity values in the seed-maps (based on SFig. 12 and SFig. 13).

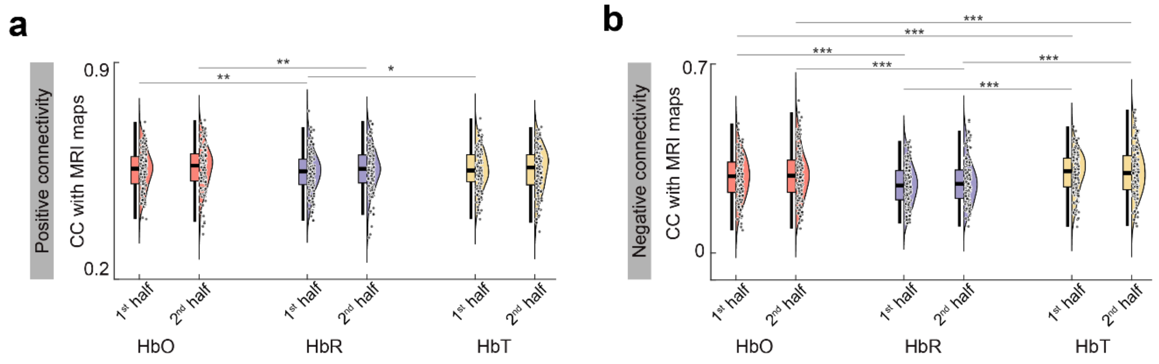

Supplementary Figure 15. (a) CC analysis of positive connections between BOLD seed maps and HbO, HbR, and HbT, using the first and second halves of the data. This analysis mirrors the approach used in Fig. 2g,h. Higher correlations were observed for HbO and HbT compared to HbR, particularly in the first half of the data. Additionally, HbO continued to show a stronger correlation than HbR in the second half as well. **(b)** Analysis of negative connections across the first and second halves of the data revealed that HbO and HbT consistently demonstrated superior correlations compared to HbR in both halves. Notably, these findings closely align with the results obtained when analyzing the full-length data, as shown in Fig. 2g-h. (*) $p < 0.05$, (**) $p < 0.01$, (***) $p < 0.001$, paired t-test. The central line within the box represents the mean, while the lower and upper box edges denote the 25th and 75th percentiles, respectively. The whiskers extend to the furthest data points within the non-outlier range.

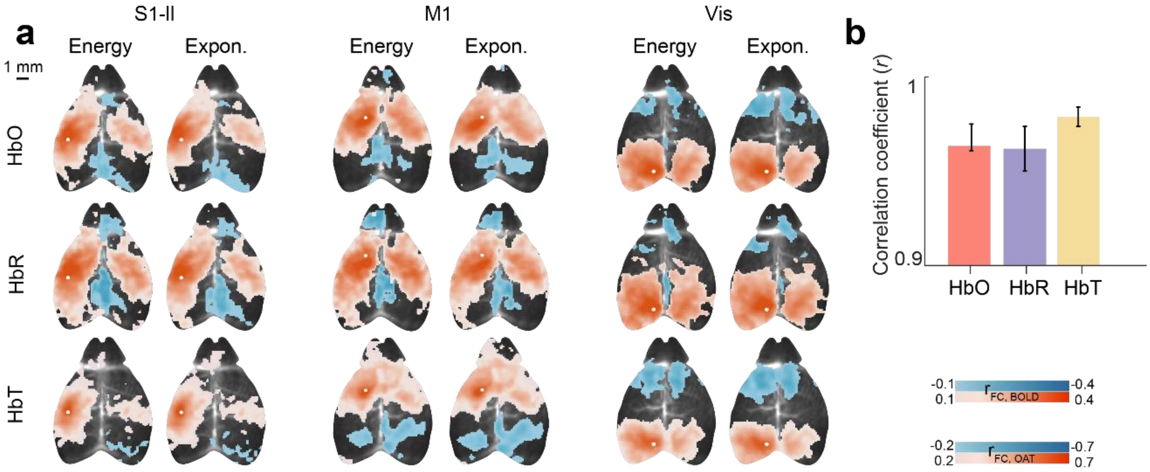

Supplementary Figure 16. (a) fOA seed-maps from a representative subject, generated using different fluence correction methods. The first method involves normalizing the optoacoustic signal by the light energy at each wavelength. The second method builds on this by incorporating an additional exponential depth-dependent fluence correction. **(b)** Pearson's correlation coefficients between seed-maps (14 maps in total) constructed using the two different fluence correction methods. S1-ll, primary somatosensory area, lower limb; M1, primary motor cortex; Vis, primary visual area. Data are presented as mean values +/- SEM.

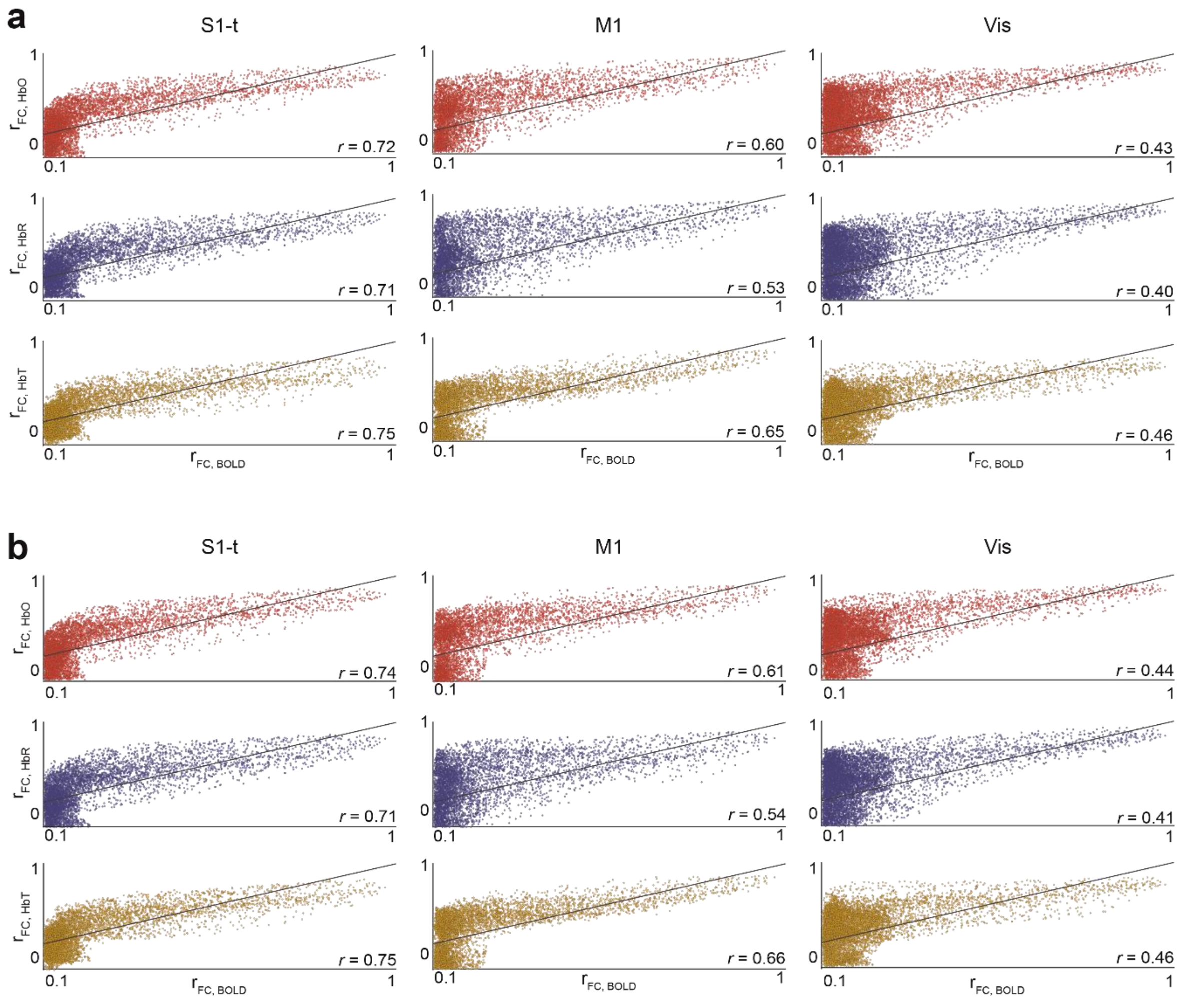

Supplementary Figure 17. Voxel-wise correlations between fOA and BOLD seed-maps constructed using the energy-based model (a) and the exponential depth-dependent model (b). S1-t, primary somatosensory area, trunk; M1, primary motor cortex; Vis, primary visual area.

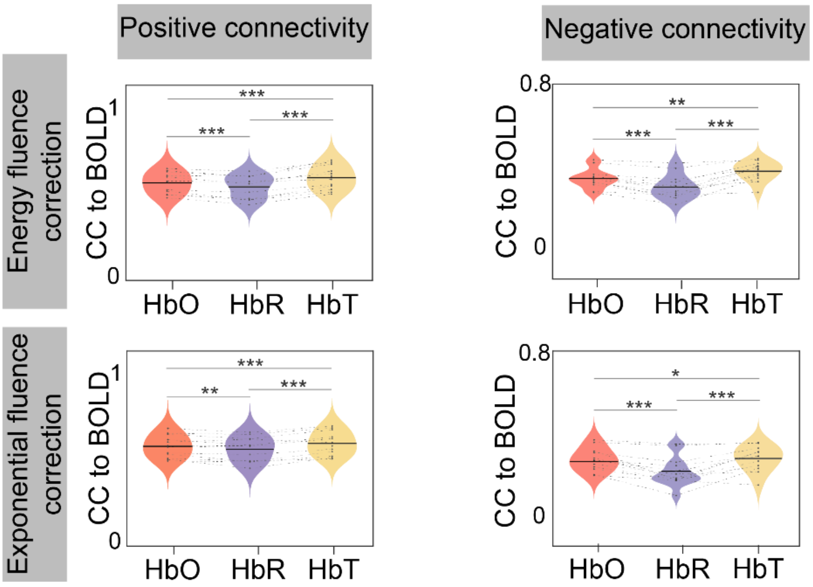

Supplementary Figure 18. Cross-correlation of fOA to BOLD seed-maps for positive and negative connections constructed using the energy-based model and the exponential depth-dependent model.

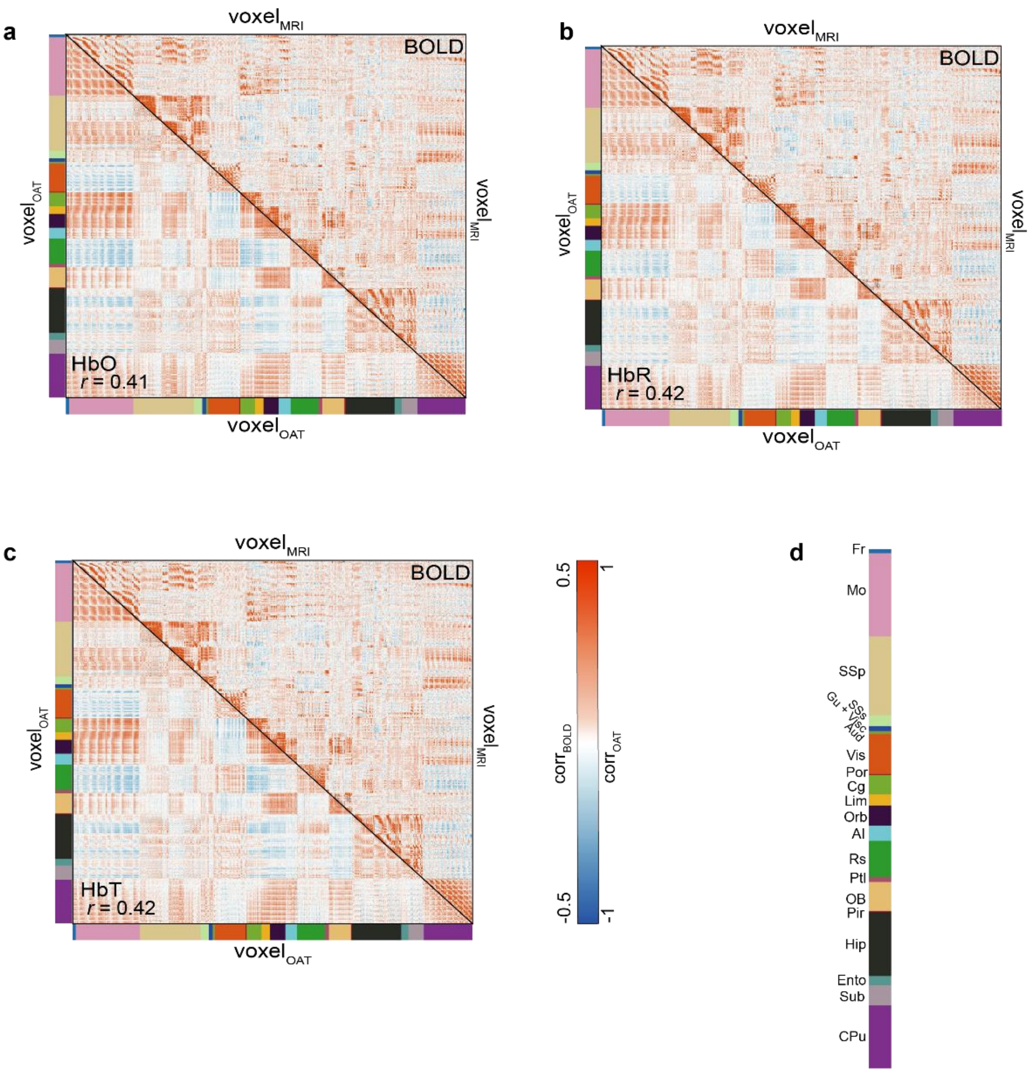

Supplementary Figure 19. Voxel-to-voxel connectivity matrices in gray matter voxels across hemodynamic components (a-c). The upper triangles display connectivity derived from BOLD imaging, while the lower triangles represent connectivity from OAT components. Pearson correlation coefficients between the BOLD matrix and the corresponding OAT matrix are shown in each panel for comparison. Panel (d) provides a color-coded representation of the brain regions where the analyzed voxels are located. Fr = frontal pole; Mo = motor areas; SSp = primary somatosensory areas; SSs = supplementary somatosensory areas; Gu = gustatory areas; Visc = visceral areas; Aud = auditory areas; Vis = visual areas; Por = postrhinal areas; Cg = anterior cingulate areas; Lim = limbic areas; Orb = orbital areas; AI = agranular insular area; Rs = retrosplenial areas; Ptl = parietal areas; OB = olfactory bulb; Pir = piriform area; Hip = hippocampus; Ento = entorhinal area; Sub = subiculum; Cpu = caudoputamen.

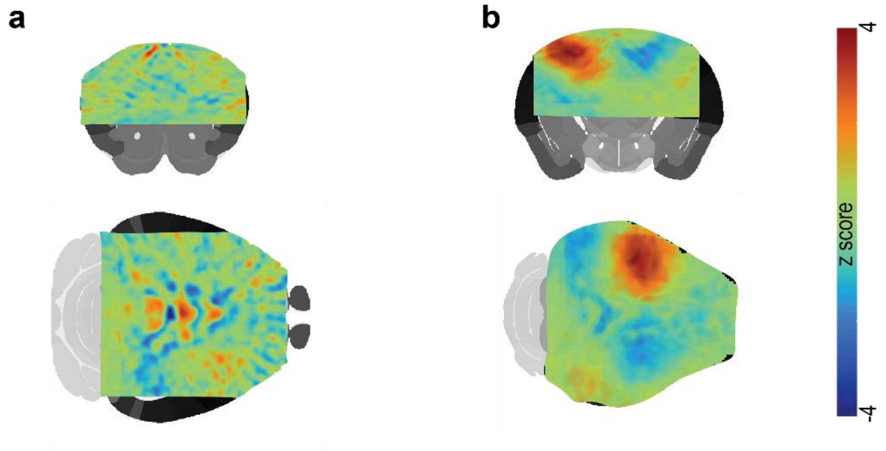

Supplementary Figure 20. Representative ICA noise components classified as reconstruction noise (a) and spurious component (b).

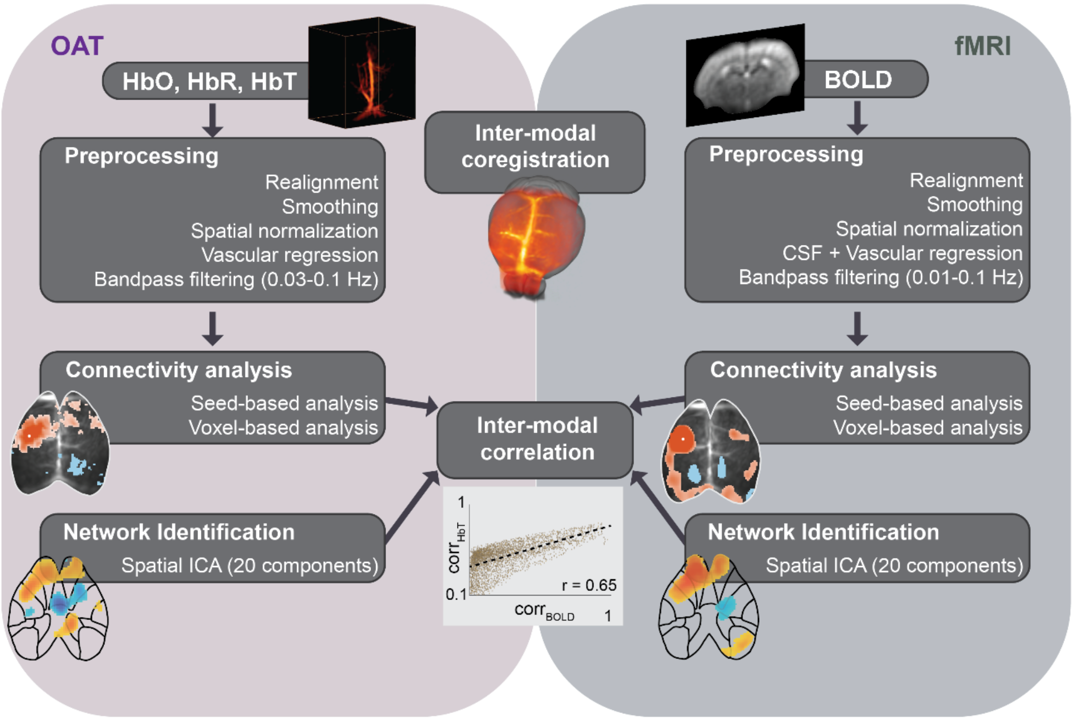

Supplementary Figure 21. Data processing pipeline of the multi-modal resting-state data. BOLD, HbO, HbR, and HbT data were preprocessed separately. Each component underwent motion correction, spatial smoothing and normalization to the Allen Common Coordinate Framework (CCFv3) following inter-modal coregistration. Functional data was temporally filtered, detrended and nuisance signals were regressed out including CSF signal, vascular signal, and motion parameters. The denoised multimodal data were then utilized to derive functional connectivity maps and elucidate intricate brain networks in mice.

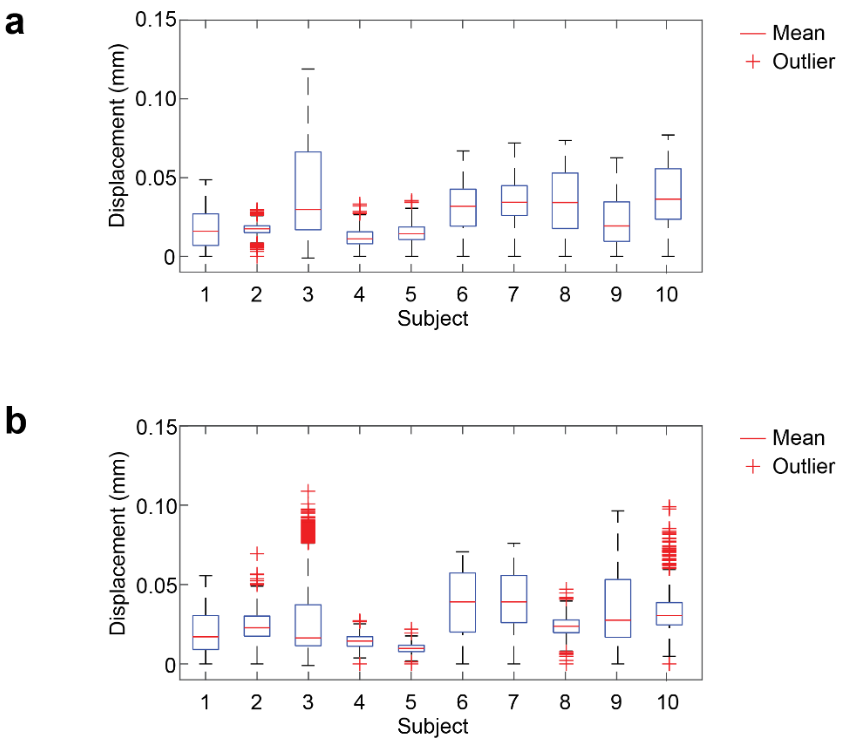

Supplementary Figure 22. Mean framewise displacement calculated per subject from OAT (a) and BOLD (b) data. The central line within the box represents the mean, while the lower and upper box edges denote the 25th and 75th percentiles, respectively. The whiskers extend to the furthest data points within the non-outlier range.

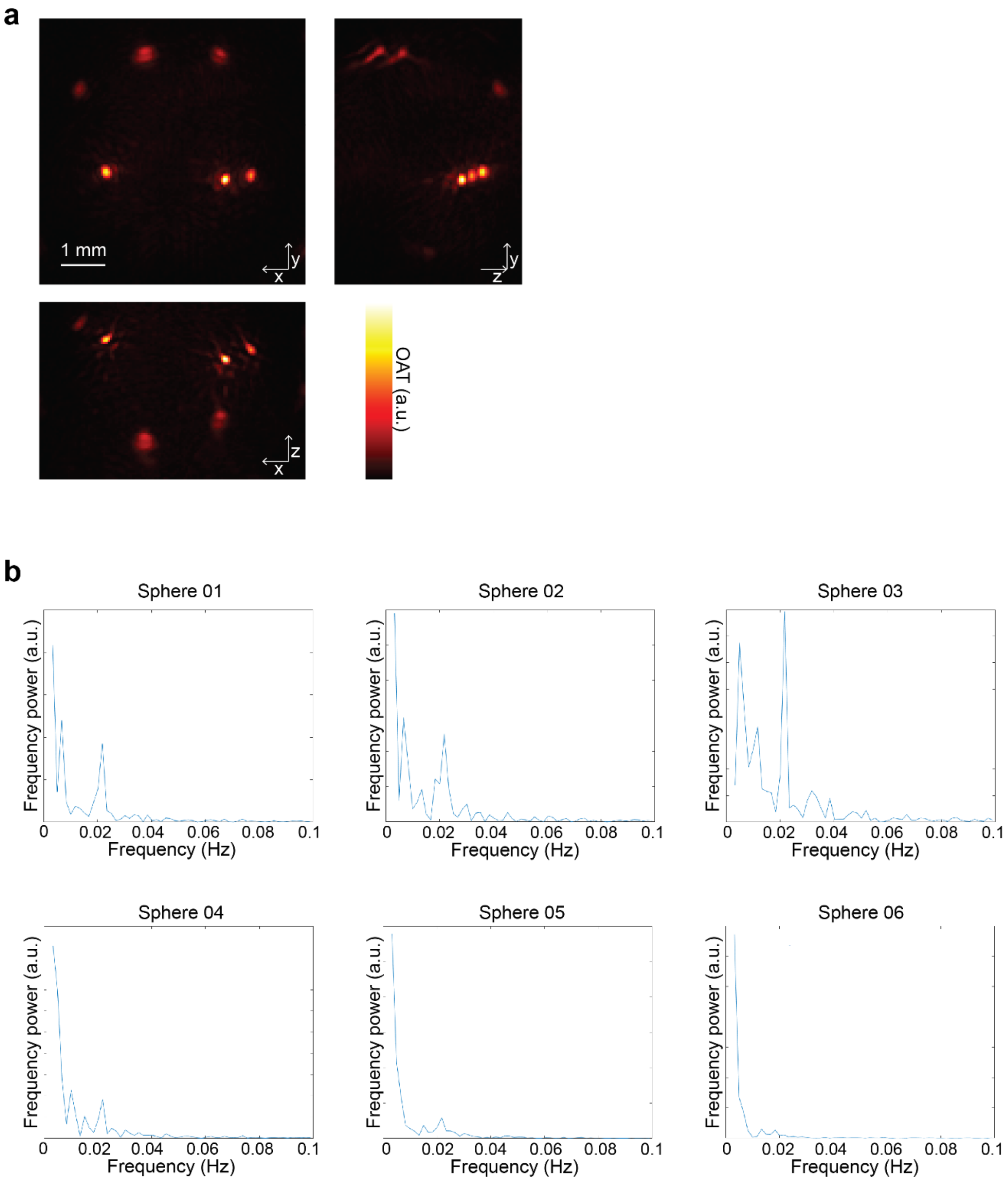

Supplementary Figure 23. Laser fluctuation signal characterization. (a) OAT image of six 100 um microspheres distributed within the FOV at 800 nm wavelength. (b) Power spectral density analysis derived from a 600-second recording at a sampling rate of 100 Hz, showing the temporal frequency components of the microspheres' optoacoustic signals.

Supplementary Table 1. Full list of regions related to Fig. 3 in the Main Text.

Cortical area	Region name
Motor areas	Primary motor area
	Secondary motor area
Primary somatosensory areas	Primary somatosensory area, nose
	Primary somatosensory area, barrel field
	Primary somatosensory area, lower limb
	Primary somatosensory area, mouth
	Primary somatosensory area, upper limb
	Primary somatosensory area, trunk
Supplementary somatosensory areas	Supplemental somatosensory area
Gustatory areas	Gustatory areas
Visceral areas	Visceral area
Auditory areas	Dorsal auditory area
	Primary auditory area
	Posterior auditory area
	Ventral auditory area
Visual areas	Anterolateral visual area
	Anteromedial visual area
	Lateral visual area
	Primary visual area
	Posterolateral visual area
	Posteromedial visual area
Postrhinal areas	Postrhinal area
Anterior cingulate areas	Anterior cingulate area, dorsal part
	Anterior cingulate area, ventral part
Limbic areas	Prelimbic area
	Infralimbic area
Orbital areas	Orbital area, lateral part
	Orbital area, medial part
Retrosplenial areas	Retrosplenial area, lateral agranular part
	Retrosplenial area, dorsal part
Anterior areas	Anterior area
